# Effectiveness of three interventions for secondary prevention of low back pain in the occupational health setting - a randomised controlled trial with a natural course control

**DOI:** 10.1186/s12889-018-5476-8

**Published:** 2018-05-08

**Authors:** J. Rantonen, J. Karppinen, A. Vehtari, S. Luoto, E. Viikari-Juntura, M. Hupli, A. Malmivaara, S. Taimela

**Affiliations:** 1 University of Helsinki, Doctoral School in Health Sciences, Helsinki, Finland; 2 Lappeenranta University of Technology, Lappeenranta, Finland; 3 Department of Occupational Medicine, South Karelia Social and Health Care District, Lappeenranta, Finland; 4 Medical Research Center Oulu, University of Oulu and Oulu University Hospital, Oulu, Finland; 5 Finnish Institute of Occupational Health, Oulu, Finland; 6 Finnish Institute of Occupational Health, Helsinki, Finland; 7 Helsinki Institute for Information Technology HIIT, Department of Computer Science, Aalto University, Espoo, Finland; 8 Department of Physical Medicine and Rehabilitation, South Karelia Social and Health Care District, Lappeenranta, Finland; 9 National Institute for Health and Welfare, Centre for Health and Social Economics, Helsinki, Finland; 10 Evalua International, Espoo, Finland; 11 Department of Orthopedics and Traumatology, Helsinki University Hospital and University of Helsinki, Helsinki, Finland

**Keywords:** LBP, Quasi-randomised design, Multidisciplinary, Graded activity, Sickness absence, Disability, RCT, Usual care, Natural course of LBP, Rehabilitation, Population based, Early management

## Abstract

**Background:**

We assessed the effectiveness of three interventions that were aimed to reduce non-acute low back pain (LBP) related symptoms in the occupational health setting.

**Methods:**

Based on a survey (*n* = 2480; response rate 71%) on LBP, we selected a cohort of 193 employees who reported moderate LBP (Visual Analogue Scale VAS > 34 mm) and fulfilled at least one of the following criteria during the past 12 months: sciatica, recurrence of LBP ≥ 2 times, LBP ≥ 2 weeks, or previous sickness absence. A random sample was extracted from the cohort as a control group (Control, *n* = 50), representing the natural course of LBP. The remaining 143 employees were invited to participate in a randomised controlled trial (RCT) of three 1:1:1 allocated parallel intervention arms: multidisciplinary rehabilitation (Rehab, *n* = 43); progressive exercises (Physio, *n* = 43) and self-care advice (Advice, *n* = 40). Seventeen employees declined participation in the intervention. The primary outcome measures were physical impairment (PHI), LBP intensity (Visual Analogue Scale), health related quality of life (QoL), and accumulated sickness absence days. We imputed missing values with multiple imputation procedure. We assessed all comparisons between the intervention groups and the Control group by analysing questionnaire outcomes at 2 years with ANOVA and sickness absence at 4 years by using negative binomial model with a logarithmic link function.

**Results:**

Mean differences between the Rehab and Control groups were − 3 [95% CI -5 to − 1] for PHI, − 13 [− 24 to − 1] for pain intensity, and 0.06 [0.00 to 0.12] for QoL. Mean differences between the Physio and Control groups were − 3 [95% CI -5 to − 1] for PHI, − 13 [− 29 to 2] for pain intensity, and 0.07 [0.01 to 0.13] for QoL. The main effects sizes were from 0.4 to 0.6. The interventions were not effective in reducing sickness absence.

**Conclusions:**

Rehab and Physio interventions improved health related quality of life, decreased low back pain and physical impairment in non-acute, moderate LBP, but we found no differences between the Advice and Control group results. No effectiveness on sickness absence was observed.

**Trial registration:**

Number NCT00908102 Clinicaltrials.gov

## Background

After the first episode of low back pain (LBP), the likelihood of a new episode clearly increases [1, 2]. The lifetime prevalence of LBP varies between 38 and 84% in the adult populations [3–5]. LBP is one of the leading causes of disability all over the world [6]. LBP related disability often leads to serious socio-economic consequences at personal, employer or societal level among the workforce [6, 7]. In order to prevent LBP from developing into a chronic, potentially disabling condition, risk based assessments and secondary prevention in the early stages of LBP have been recommended [5, 8]. However, high quality studies are still lacking, especially among the working-age population [9–11].

A great deal of prior randomised controlled trials (RCT) about LBP management in the occupational health (OH) setting have focused on facilitating return-to-work from sickness absence [12–17], i.e., tertiary prevention. Multidisciplinary rehabilitation still holds the best evidence in relieving chronic LBP related symptoms and facilitating return-to-work [18, 19]. Only few trials have evaluated the effectiveness of interventions among non-sick-listed workers in the OH setting [20, 21]. Suni et al. [20] included male railway workers after a questionnaire survey about LBP related symptoms and obtained positive results with a neuromuscular physical training and counselling program. In our previous RCT, both male and female employees (aged from 18 to 56) were recruited based on an employee survey. Eligible employees reported moderate level LBP, but were not sick-listed prior to randomisation [21]. Two active interventions, progressive exercises and multidisciplinary rehabilitation, reduced pain and physical impairment at 1 year in comparison to OH physician’s advice [21]. Although these results show that active interventions were effective in comparison to self-care advice, it is not clear whether early management (population-based screening, subgrouping and activity based intervention), in general, should be recommended for non-sick-listed employees. The main questions about secondary prevention of LBP remain *for whom, how and when* preventive actions should be administered.

We performed a controlled, longitudinal intervention trial in the OH setting, alongside with our previous RCT. Participants that reported non-acute, moderate level LBP were later randomised into three intervention arms and compared to a parallel control group that represents the natural course of LBP. Effectiveness of the interventions was assessed in comparison to the natural course of LBP by using questionnaire outcomes at 2 years and sickness absence outcomes at 4 years.

Our study hypotheses were as follows:

1. Based on a LBP specific employee survey, it is feasible to select a cohort of employees that represent increased risk of disabling LBP but are still able to work.
2. Three LBP specific interventions (multidisciplinary rehabilitation, progressive exercises and self-care advice) will reduce low back symptoms, related disability and sickness absence in comparison to natural course of LBP among the employee cohort.

## Methods

### Study design and setting

This study is a part of a larger longitudinal cohort study which was executed in the occupational health setting. All employees (*N* = 2480) in a forestry company were invited to respond to a postal survey that included questions on LBP history, LBP intensity, physical impairment (PHI) and pain related fear. Based on their responses, employees (*N* = 1754, response rate 71%) were assigned into three main categories: “no” symptoms, “some” LBP related symptoms and LBP symptoms that “potentially hamper work”. The cohort includes two embedded RCTs and two respective control groups, based on random sampling, representing the natural course of LBP. The main results of both RCTs have already been published elsewhere [21, 22].

### Study aim and ethics

In the present study, we assessed long-term effectiveness of three interventions in comparison to the natural course of LBP alongside with our previous RCT [21] on subjects with low back symptoms that potentially hamper work, later labelled as “moderate” LBP as described in Table 1.

**Table 1:** Inclusion and exclusion criteria for the study are based on the data of the employee survey

Inclusion criteria	Exclusion criteria
Permanent employment	No permanent employment or retirement within the time span of the study (2 years)
Male or female, age ≤ 56 years	Age > 56 years
LBP^a^ during last week ≥35 mm with Visual Analogue Scale	LBP^a^ during last week< 35 mm with Visual Analogue Scale
At least one of the following criteria is fulfilled during the last 12 months:	Presence of any of the following conditions: pregnancy, acute nerve root compression symptoms, suspicion of a malignant tumour, recent fracture or severe osteoporosis or any other specific disease that might prevent participation in the follow- up
1. “Sciatica” - LBP that has radiated below the knee level 2. “Prolongation” - LBP that prolonged for two weeks or more 3. “Recurrency” - LBP has recurred two times or more 4. “Work absence” - LBP related sickness absence	

^a^LBP Low back pain

The South Karelian Central Hospital Research Ethics Board approved the study and it was performed according to the Declaration of Helsinki.

### Participants

The inclusion and exclusion criteria for the study are presented in Table 1.

**Fig. 1:**
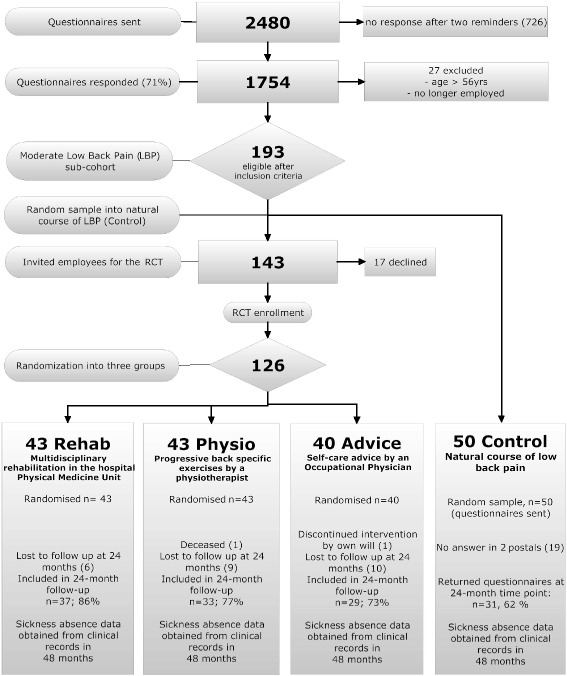
The flow chart of the study and the number of participants at different stages of the trial

Eligible employees (*n* = 193) were included in the sub-cohort “moderate LBP”. At first, we extracted a random sample of 50 employees from the sub-cohort “moderate LBP” and defined them as the control group (Control), representing the natural course of LBP (Fig. 1). The remaining employees (*n* = 143) were invited to participate in the RCT, of which 17 declined. After an informed consent, 126 participants were randomised into three intervention groups: physical medicine outpatient unit in a hospital (Rehab, *n* = 43), progressive back exercises in an outpatient clinic (Physio, n = 43), and self-care advice by the OH physician, (Advice, *n* = 40) (Fig. 1). Employees that were selected in the Control group were followed up for 2 years, similarly as the RCT participants.

There were no differences in the baseline characteristics between the pooled intervention groups and the Control group (Table 2).

**Table 2:** Baseline characteristics of the study participants. Means (SD = standard deviation) or percentages when applicable

Characteristics	Rehab (*n* = 43)			Physio (n = 43)			Advice (*n* = 40)			Control (*n* = 50)			
	%	mean	SD	%	mean	SD	%	mean	SD	%	mean	SD	*p*
Demographic features													
Age, years	.	45	9	.	44	8	.	45	7	.	46	7	0.44
Male, %	65	.	.	72	.	.	68	.	.	60	.	.	0.30
High School diploma/vocational degree, %	67	.	.	56	.	.	58	.	.	70	.	.	0.23
Health related features													
Duration of LBP, years	.	13	9	.	10	9	.	14	9	.	11	9	0.51
Smoking, %	31	.	.	40	.	.	40	.	.	23	.	.	0.14
Work-related features													
Blue collar worker, %	74	.	.	77	.	.	90	.	.	84	.	.	0.56
Shift worker^a^, %	43	.	.	28	.	.	39	.	.	32	.	.	0.68
Physical workload (1–5)^b^	.	3	1	.	3	1	.	3	1	.	3	1	0.48
Mental workload (1–5)^b^	.	3	1	.	3	1	.	3	1	.	3	1	0.07
Work ability (0–10)^c^	.	7	2	.	7	2	.	7	2	.	7	2	0.84
Total SA days/previous year	.	16	23	.	21	28	.	19	26	.	22	49	0.62
Total SA periods/previous year	.	4	5	.	4	4	.	4	4	.	4	5	0.99
Low back specific SA days/previous year	.	6	14	.	9	25	.	9	22	.	9	41	0.92
Outcome variables at baseline													
LBP intensity, LBP (0–100)	.	60	17	.	55	14	.	60	18	.	60	18	0.47
Physical impairment, PHI (0–18)	.	8	5	.	8	5	.	8	5	.	7	5	0.19
Pain related fear, FABQ (13–78)	.	38	14	.	38	12	.	37	12	.	35	12	0.26
FABQ work, FABQw (6–36)	.	18	7	.	16	7	.	16	7	.	16	7	0.61
FABQ physical activity, FABQph (4–24)	.	13	5	.	14	5	.	13	5	.	12	4	0.09

^a^two-shift or three-shift work

^b^self rated workload: 1 = very heavy, 2 = moderate, 3 = intermediate, 4 = rather light, 5 = very light

^c^range 0–10 from the lowest possible work ability (0) to the best possible work ability (10)

The three intervention groups were pooled for the comparison with the Control group and P denotes the p-value of the comparison. Range (when applicable) is presented after the variable name in the parenthesis

*Rehab* outpatient rehabilitation at the physical medicine unit, *Physio* progressive back-specific exercises, *Advice* self-care advice by an OH physician, *Control* Natural course control group, *SA* sickness absence, *LBP* low back pain, *VAS* Visual Analogue Scale, *PHI* physical impairment by the Roland-Morris Disability Questionnaire (0 to 18), *FABQ* Fear Avoidance Beliefs questionnaire, *FABQw* Fear Avoidance beliefs questionnaire, work subscale, *FABQph* Fear Avoidance beliefs questionnaire, physical activity subscale

### Randomisation and blinding

The Control group, 50 employees, was extracted from the original, moderate LBP sub-cohort of 193 employees as a random sample by means of a computer program. The remaining 143 employees of the sub-cohort were then invited to participate in the baseline visit for the RCT.

The randomisation scheme for the RCT was prepared by an independent biostatistician using a computer-generated randomisation table. To prevent unequal randomisation of subjects by age and gender in the treatment arms, scripted four-digit identification codes were sorted by gender and age (≤45 years, > 45 years), resulting in four strata. Block randomisation (with blocks of 15) was applied to ensure equal group sizes within each stratum. Based on the randomisation scheme, before the start of the study, a research assistant prepared sealed envelopes containing either a referral to the Rehab group, the Physio group or the Advice group. During the baseline visit for the RCT, after signing the informed consent form, each employee opened a sealed envelope containing the group allocation. The research personnel were not able to identify the workers or their group assignments before randomisation.

Due to the nature of the interventions, the participants and OH professionals could not be blinded of their group assignments. Sickness absence and questionnaire data were gathered and entered into the computer by separate research assistants, ensuring blinded analysis of the data by the researchers.

### Interventions

All participants were free to use all health care services during the study interventions and follow-up, as usual.

The baseline visit, including the randomisation, lasted about 60 min in both active intervention groups, but in the Advice group, the first visit took an extra 20-min time because of the Back Book® information session.

After the randomisation visit, there were four scheduled follow-up visits in all three intervention groups at 3, 6, 12, and 24 months. Employees were instructed to fill out their follow-up questionnaires during the preceding week prior to their respective visit date. Intervention groups were comparable regarding the follow-up intervals, visit activity and the 30 min’ time spent at the follow-up visits. Detailed description of RCT study visits and interventions have been published elsewhere [21].

The company occupational health services unit operated as usual during the study period. Information about the study was provided regularly in the company magazine, bulletin boards, and intranet.

#### Rehab – Physical medicine unit

The multidisciplinary LBP rehabilitation in the Rehab group was carried out at the local hospital’s physical medicine outpatient unit. First, the program included exercise sessions 2–3 times per week about one hour each over 3 weeks, followed by an intensive 3-week period of 6 h per day, 5 days per week. The whole intervention took about 111 h in 6 weeks and was performed in 5 groups consisting of 8 to 10 individuals each.

#### Physio – Progressive back exercises

A graded activity program was carried out individually for each patient in the Physio group under supervision of a specially trained physiotherapist in a physiotherapy outpatient clinic. The intervention included a one-hour session twice or three times per week, integrated with each participant’s daily program over a period of 12 weeks. The whole intervention took about 24 to 36 h.

#### Advice – Self-care advice by an OH physician

In the first study visit, participants received the Back Book® [23] booklet and their occupational physician also explained the contents of the booklet for them individually. The Back Book® contents follow the general LBP guidelines by emphasising benign nature and good prognosis of non-specific LBP and suggesting rapid return to normal activities [23].

#### Control - natural course of LBP

The participants in the Control group were not invited to any study visits. They only received the follow-up questionnaire once, about 2 years after the employee survey. One reminder was sent to those who did not respond to the first postal survey. No other contacts or interventions were performed in the Control group on behalf of the study personnel. Thirty-one (31) responses (62%) were received.

### Outcome measures and data collection

The questionnaire outcomes were evaluated at the 24-month time-point in comparison to the Control group. Baseline measurements originate from the employee survey.

All cause (=total) sickness absence data were obtained from the electronic medical records of the occupational health services. Based on the secondary prevention focus in this study, we accumulated all cause sickness absence days and periods over 48 months in order to evaluate long-term effectiveness and also to reduce the skewness that was related to sickness absence data.

The individual inclusion date serves as a starting point for the sickness absence follow-up until 4 years. In the intervention groups, inclusion date is the date of randomisation and in the Control group, postal date of the employee survey. Sickness absence data are comprehensive and highly reliable because they are based on the administrative payroll system of the employer. There were no missing values in the sickness absence data, either. However, typically sickness absence data is highly skewed, over-dispersed with zeros and includes some extremely high values. Long-term non-LBP-specific sickness absence episodes (over 30 days each), that originate e.g., from severe diseases and sequels of other than low-back specific injury, generally interfere with the analysis but, most likely, are not connected with the effectiveness of the interventions. Because each sickness absence episode in our data also holds a specific ICD-10 diagnosis code [24], we were able to identify and exclude all non-LBP-specific episodes that were longer than 30 days from the original data. In 4 years, excluded SA periods affected about 26–28% of the participants in all four study groups, with no difference in sickness absence periods or days between the groups. In summary, our cumulative sickness absence days and periods in 4 years include all LBP-specific sickness absence days and periods, regardless of their length and all other cause sickness absence that are less than 31 days. The 30-days’ cut-off limit was chosen arbitrarily before conducting any analyses.

#### Primary outcome measures

The follow-up questionnaire at the 24-month time point included previously validated and widely used outcome measures as follows: physical impairment (PHI) with Roland-Morris 18-item scale [25, 26], LBP intensity during the preceding week (VAS, Visual Analogue Scale, range 0-100 mm) [27, 28] and quality of life with 15-D quality of life questionnaire score (QoL, range 0–1) [29, 30]. The accumulated number of sickness absence days over the 4-year follow up were collected from the OH registers that synchronise with the employer’s payroll system.

#### Secondary outcome measures

The follow-up questionnaire at the 24-month time point included the following, previously validated and widely used outcome measures: Disability (OSW, sum value of the Oswestry Disability Index) [31, 32], pain related fear (FABQ; Fear Avoidance Beliefs Questionnaire) [33], subscales FABQ work (FABQ_w_, range 6–36) and FABQ physical activity (FABQ_ph_, range 4–24) [33–35]. The accumulated number of sickness absence periods over the 4-year follow up were collected the same way as the sickness absence days.

### Power calculation

Power calculations were performed at the design phase of the study, based on the group difference in LBP intensity (VAS). The standard deviation was expected to be 15 units (mm). Calculations showed that differences in LBP intensity of 10 mm between groups will be detectable with 80% power in two-tailed tests with a significance level of 0.05 for a sample of 40 employees in each group.

### Statistical analyses

All statistical analyses were performed at the employee level, according to the intention-to-treat principle. Baseline characteristics were compared using descriptive statistics, in which the intervention groups were pooled for the comparisons to Control group.

About 29% of the questionnaire follow-up data were missing, but equally in all groups and mostly due to some totally missed follow-up visits. We are not aware of any systematic reasons or motives that would explain the missed visits or non-response in any of the study groups. All participants in this study had an equal opportunity to attend follow-up visits during their working hours and they were also offered several alternatives to change their visit schedule. Some participants sent their questionnaire data to the study personnel if they were not able to attend personally. Hence, based on our best knowledge, missing questionnaire data is missing at random. Missing values in the questionnaire-based outcome variables were imputed with the multiple imputation method [36] of the IBM SPSS Statistical Package version 24.0 for Windows ® (IBM Inc., Chicago, IL, USA). The following items were used as determinants when conducting the missing data imputation: age, gender, marital status, education, smoking, lifetime duration of LBP, self-assessed health status, working status, shift work, physical workload, mental workload, self-assessed work ability, job satisfaction, physical impairment, LBP, pain-related fear, all cause sickness absence 12 months prior to the employee survey and all cause sickness absence over the first year.

The effectiveness of an intervention was primarily estimated with the group difference of all outcome variables in comparison to Control (i.e. Rehab vs. Control; Physio vs. Control, and Advice vs. Control).

As regards the questionnaire variables, primary (PHI, LBP intensity, QoL) and secondary outcome variables (disability, FABQ, FABQ_w_, FABQ_ph_) were assessed at 24 months after the employee survey. The 95% confidence intervals (CI) for the mean differences were computed using the generalized linear model ANOVA where the respective baseline values were used as covariates (when appropriate). Effect sizes of all outcomes, in all study group comparisons, were estimated with Cohen’s d [37].

For each calendar year, sickness absence data was highly skewed, including both an excess number of zero values and some very high values. However, accumulation of the sickness absence data in 4 years resulted into a smaller proportion of zero values. We tested several linear and non-linear models and hierarchical structures. Previous year sickness absence data, i.e. one-year sickness absence days and periods before the study were used as covariates, respectively. Finally, the best model for the sickness absence distribution in the present study was achieved by the negative binomial model with a logarithmic link function.

### Lost to follow-up

By the end of the follow-up, one participant from the Advice group left the study due to personal reasons, but granted permission to use the data. At the end of the two-year follow-up, 6 participants of the Rehab group, 10 participants of the Physio group and 11 participants of the Advice group failed to return their questionnaires, resulting in missing data. One participant from the Physio group deceased 3 months before the final visit. Regarding the intervention groups, 99 participants (Rehab, *n* = 37; Physio, *n* = 33 and Advice, *n* = 29) continued to the final visit, resulting in participation rates 86%, 77% and 73%, respectively. In the Control group, data was available for 31 (62%) participants.

## Results

### Employee flow

Figure 1 presents the flow of the participants in this trial.

### Primary outcomes

The results are shown in Tables 3, 4 and 5.

**Table 3:** Results of the questionnaire outcome variables after 2 years’ follow-up. The primary and secondary variables in all study groups and the group comparisons between the intervention groups (Rehab, Physio, Advice) and Control group. Mean, standard deviation (SD), mean difference (MD), 95% confidence interval (95% CI), *p*-value of the group comparison

Outcomes / analysis	Rehab (43)		Physio (43)		Advice (40)		Control (50)		Rehab vs. Control			Physio vs. Control			Advice vs. Control		
	mean	SD	mean	SD	mean	SD	mean	SD	MD	95% CI	*p*	MD	95% CI	*p*	MD	95% CI	*p*
Primary outcomes																	
Physical impairment	4.7	4.5	4.7	4.4	5.9	4.8	7.4	4.4	**−3**	−5 to − 1	0.00	**−3**	−5 to − 1	0.00	**−2**	−3 to 0^a^	0.07
LBP intensity	27	19	29	20	32	23	40	26	**−13**	−24 to − 1	0.03	−13	−29 to 2	0.08	−10	−27 to 8	0.24
Quality of Life - QoL	0.832	0.137	0.841	0.141	0.795	0.136	0.771	0.145	**0.06**	0.00 to 0.12	0.04	**0.07**	0.01 to 0.13	0.02	0.02	−0.03 to 0.08	0.42
Secondary outcomes																	
Pain related fear - FABQ	33	15	35	13	40	16	41	13	**−8**	−14 to − 2	0.01	−5	−12 to 1	0.09	−1	−7 to 6	0.82
Pain related fear - FABQ_w_	15	7	16	8	18	8	19	8	**−5**	−8 to − 1	0.01	**−4**	−7 to 0	0.03	−1	−5 to 2	0.49
Pain related fear - FABQ_ph_	11	5	12	5	13	6	13	4	**−2**	−4 to 0^a^	0 06	−1	−3 to 2	0.57	0	−2 to 3	0.76
Disability - OSW sum	8	7	9	8	12	8	14	8	**−6**	−10 to −2	0.00	**−5**	−9 to 0	0.03	−2	−6 to 2	0.60

Analysis includes 176 participants in Rehab (43), Physio (43), Advice (40) and Control (50) groups

Physical impairment by the Roland-Morris 18-item Disability Questionnaire (range 0–18), *FABQ* Fear Avoidance Beliefs questionnaire, sum variable, *FABQ*_*w*_ Fear Avoidance beliefs questionnaire, work subscale, *FABQ*_*ph*_ Fear Avoidance beliefs questionnaire, physical activity subscale, *LBP* low back pain with Visual Analogue Scale (VAS, range 0-100 mm), *QoL* health related quality of life, *OSW* Oswestry Disability scale (sum value) ^*a*^*p*>0.05

**Table 4:** The number of accumulated sickness absence (SA) days and periods in 4 years^a,b,c^. Means, mean differences (MD) and 95% confidence intervals (95% CI)

Outcomes	Rehab		Physio		Advice		Control		Rehab vs. Control			Physio vs. Control			Advice vs. Control		
	mean	95% CI	mean	95% CI	mean	95% CI	mean	95% CI	MD	95% CI	*p*	MD	95% CI	*p*	MD	95% CI	*p*
Primary Outcome																	
SA days	67	50 to 91	74	54 to 99	84	61 to 114	72	55 to 96	−5	−34 to 24	0.73	1	−29 to 31	0.94	11	−22 to 44	0.51
Secondary Outcome																	
SA periods	8	6 to 11	12	9 to 17	15	11 to 21	13	10 to 17	**−5**	−10 to 0	0.03	−1	−6 to 5	0.84	2	−4 to 8	0.47

^a^Main analysis includes 176 participants in Rehab (43), Physio (43), Advice (40) and Control (51) groups

^b^Analyses were calculated with IBM SPSS 24 version’s Generalised linear models Negative binomial with loglink procedure

^c^SA days and periods during one year before the intervention were used as covariates, respectively

**Table 5:** Effect sizes of the primary and secondary outcomes in all study group comparisons according to Cohen’s d^a^

Analysis	Effect size (d)		
	Rehab vs. Control	Physio vs. Control	Advice vs. Control
Primary outcomes			
Physical impairment (PHI)	**0.7**	**0.7**	0.4
LBP intensity (VAS)	**0.6**	**0.6**	0.4
Quality of Life (QoL)	0.4	**0.5**	0.2
Sickness absence^b^ days	0.1	0.0	0.1
Secondary outcomes			
Pain related fear - FABQ	**0.6**	0.4	0.1
Pain related fear - FABQ_w_	**0.6**	**0.5**	0.2
Pain related fear - FABQ_ph_	0.4	0.1	0.1
Disability - OSW sum	**0.8**	**0.6**	0.3
Sickness absence^b^ periods	0.4	0.0	0.2

^a^Cohen’s d effect size is interpreted as follows: d < 0.5 small effect size; 0.5 < d < 0.8 medium; 0.8 < d < 1.2 large; d > 1.2 very large effect size. Medium or larger effect sizes are bolded

^b^Sickness absence = accumulated, all cause sickness absence during two years

*FABQ* Fear Avoidance Beliefs questionnaire, sum variable, *FABQw* Fear Avoidance beliefs questionnaire, work subscale, *FABQph* Fear Avoidance beliefs questionnaire, physical activity subscale, *LBP* low back pain, *QoL* health related quality of life, *OSW* Oswestry Disability scale (sum value)

#### Questionnaire outcomes – Physical impairment, low back pain intensity and quality of life at 24 months

The mean difference in physical impairment (PHI) at 2 years between the Rehab Group and the Control group was − 3 [95% CI -5 to − 1] and between the Physio group and the Control group − 3 [− 5 to − 1], in favour of the active  interventions.

The mean difference in LBP intensity at 2 years between the Rehab Group and the Control group was − 13 mm [− 24 to − 1], in favour of the Rehab intervention and between the Physio group and the Control group − 13 [− 29 to 2].

The mean difference in health-related quality of life (QoL) at 2 years between the Rehab Group and the Control group was 0.06 [0.00 to 0.012] and between the Physio group and the Control group 0.07 [0.01 to 0.13], in favour of the active  interventions.

No differences in PHI, pain intensity or QoL were found between the Advice group and Control group (Table 3).

Effect sizes of PHI and LBP intensity were medium (0.6 to 0.7) in Rehab vs. Control and effect sizes of PHI, pain intensity and QoL were also medium (0.5 to 0.7) in Physio vs. Control. Other effect sizes were small (Table 5).

#### All cause, cumulative sickness absence days over 4 years

In 4 years, the total number of accumulated sickness absence days in the Rehab, Physio, Advice and Control groups were 3223, 3611, 3819 and 4602 days, respectively (Table 4). None of the three interventions (Rehab, Physio, Advice) were effective in comparison to Control group in terms of total, cumulative sickness absence days in 48 months: Mean differences were − 5 days [95% CI -34 to 24], 1 [− 29 to 31] and 11 [− 22 to 44], respectively (Table 4). Effect sizes were small (Table 5).

### Secondary outcomes

#### Questionnaire outcomes – Disability, pain related fear and its subscales in 24 months

The mean difference in disability (OSW) between the Rehab group and the Control group was − 6 [− 10 to − 2] and between the Physio group and the Control group − 5 [− 9 to 0], in favour of the interventions.

The mean difference in the work subscale of pain related fear (FABQ_w_) between the Rehab group and the Control group was − 5 [− 8 to − 1] and between the Physio group and the Control group − 4 [− 7 to 0], in favour of the interventions.

The mean differences between the Rehab group and the Control group were, in the physical activity subscale of pain related fear (FABQ_ph_) -2 [− 4 to 0] and in the total FABQ scale − 8 [− 14 to − 2], in favour of the Rehab intervention.

No differences in disability or fear-related outcomes were found between the Advice group and Control group (Table 3).

Effect sizes of OSW, FABQ and FABQ_w_ were from medium to large (between 0.6 and 0.8) in Rehab vs. Control and effect sizes of OSW and FABQ_w_ were medium (0.5 to 0.6) in Physio vs. Control (Table 5).

#### All cause, cumulative sickness absence periods over 4 years

In 4 years, the total number of accumulated sickness absence periods in the Rehab, Physio, Advice and Control groups were 434, 614, 702 and 740 periods, respectively.

The mean difference between the Rehab group and the Control group was − 5 periods [− 10 to 0], between the Physio group and Control group − 1 period [− 6 to 5] and between the Advice group and Control group 2 periods [− 4 to 8], respectively (Table 4). Effect sizes were small.

### Adverse effects

No adverse events were reported during the interventions.

## Discussion

### Main findings

In comparison to natural course of LBP, both active interventions decreased physical impairment and increased the quality of life at 2 years. Pain intensity decreased in the Rehab group, but we found no effectiveness in total sickness absence days in any of the group comparisons. Patient information by an occupational physician was not effective in comparison to natural course of LBP. Our study shows that a simple questionnaire is feasible in selecting for whom, and the two active interventions were effective in determining how secondary prevention of disabling LBP may be administered.

### Strengths and weaknesses of the study

We performed a controlled, quasi-experimental study for the secondary prevention of LBP among non-sick-listed employees. The main strengths of this study are at the pragmatic and secondary preventive study design, comprehensive sickness absence data and a real world occupational health setting with the presence of a natural course control group.

The study base (2480 employees) aptly represents the general distribution of Finnish work-force (age, gender, socio-economic class, physical and mental workload). Although all participants reported non-acute, yet moderate level and chronic LBP in the screening phase, they were all primarily able to work. The control group was selected as a random sample from the same cohort of eligible employees with the intervention arms, prior to the randomisation procedure. Based on the inclusion criteria, we suggest good generalisability of the results. The study setting also fits well to our secondary preventive purpose.

The quasi-experimental study design may be considered as a weakness. Although the Control group was a random sample, the intervention groups were randomised separately. One of our main goals was not to interfere the participants in the Control group with any study visits. Moreover, they were sent only one follow-up questionnaire after 2 years.

The follow-up rates were satisfactory in all groups. In contrast with the good follow-up activity in the intervention groups (73–86%), the response rate in the Control group was somewhat lower (62%), which could potentially indicate selective participation and cause bias. However, there was no difference in the baseline variables between the intervention groups and Control, Advice and Control or with the basic characteristics of respondents and non-respondents in the Control group. Therefore, we believe that neither the advice nor the follow-up visits in the intervention groups affected the outcomes per se*.* It also seems, that lower response rate in the Control group did not actually hamper the comparability of the groups.

### Methodological considerations

Both Rehab and Physio were well established in clinical practice for chronic or subacute LBP patients [21] at the time of designing this study. Therefore, we considered these interventions as suitable for the secondary prevention of low back symptoms as well.

All questionnaire-based outcome variables in this study have previously been used in several other intervention studies [18, 19].

Instead of choosing LBP specific sickness absence as an outcome measurement in this study, we chose all cause sickness absence because it is generally considered as a measure of health in the working population when health is understood as a mixture of social, psychological and physiological functioning [38, 39]. Recorded sickness absence data have several advantages: the quality of the data in terms of coverage, accuracy and consistency over time is superior to that achievable via self-reports. Our sickness absence data were skewed and included several outliers, which is typical in the analyses of sickness absence [39, 40].

Multiple imputation is a modern method to impute missing values in longitudinal intervention studies [41, 42]. If only original data were analysed, substantial parts of the data would have been left out from the analyses and the risk of losing essential information would rise. However, our study results (by using multiple imputation) were consistent with the results based on the original data (data not shown).

### Comparison with previous studies

There are only few comparable studies that have randomised employees with non-acute LBP into active exercise interventions in the OH setting [9, 10]. A recent systematic review on secondary prevention of LBP found low-quality evidence for exercise alone and moderate-quality evidence for exercise with education to lower the risk of future LBP episodes among employees [11]. However, most of the earlier studies were not comparable to our study, because of the differences in patient recruitment strategy, gender, age or profession.

A previous Cochrane review has concluded that LBP specific physical exercise, alone or together with psychosocial intervention or pain management was effective in reducing both clinical symptoms and sickness absence in chronic LBP [19]. Also some recent studies [13, 14, 43, 44] have included patients that were initially more symptomatic than the subjects in our study. Recruitment in these prior studies was based on work absence records or back clinic consultations [8, 18, 45, 46]. These studies were also focused at increasing return-to-work from sickness absence. Because of higher symptom level, different recruitment strategy and a large variety of interventions, comparisons between these studies are complex.

In our previous RCT [21], both active interventions (Rehab and Physio) reduced short-term LBP and improved long-term quality of life (Physio) in comparison to low back specific information that was delivered by an occupational physician (Advice). Rehab also decreased the incidence of new sickness absence periods (all cause), but not earlier than after 3 to 4 years [21]. The results of the present study are consistent with our earlier findings.

Although quality of life did increase in both active intervention arms, results in several other studies have shown mixed effectiveness in QoL [17].

Hay et al. introduced an effective strategy for the early management of LBP in the UK. Participants were distributed into low-, medium- and high-risk LBP subgroups in the primary care [47]. Stratified interventions that were targeted in each subgroup accordingly, decreased short-term physical impairment in the medium- and high-risk groups more than non-stratified best practice. Patients were included in the study, if they consulted their primary care physician [48]. Despite of different recruitment strategy, there are many similarities with the design in comparison to our study. Importantly, subgrouping patients into pre-defined risk groups and delivering active interventions accordingly resulted in consistent, positive results that were comparative with our study results [47].

Although patient information that was delivered by the OH physician was not effective in moderate level low back symptoms, our previous studies have concluded that simple patient information delivered by OH nurse is effective [22, 49] and cost-effective [49] in mild level LBP.

In order to reduce recurrent, sub-acute and chronic LBP at personal, workplace or community level, current evidence already suggests targeted and stratified approach [48] but also the ability to adopt multiple management strategies. Especially, when we are dealing with heterogenic patient groups that are generally met in the primary or OH care, successful management strategy includes exercise interventions, holistic assessment [8], advice and patient information [50], return to work procedures [51, 52] and also ergonomic interventions when needed [53, 54].

### Clinical significance of the study

Our pragmatic study was performed in the OH centre of a large forestry company in Lappeenranta, Finland. The OH centre was situated near the factory area, similarly as any other primary care unit that is serving its customers. Participants were men and women, between 24 and 56 years old, who reported various physical and mental demands in their work. The participants’ mean total pain level was 59 mm (SD 17 mm; VAS: 0 – 100 mm) and physical impairment was 8 units (SD 5 units; Roland-Morris: 0–18 units) at baseline. Such individuals are at risk of recurrent, progressing LBP [55], representing 11% of the total number of survey respondents. Although their working ability was already reduced, they were still working at the inclusion, suggesting that the target group was suitable for secondary prevention of LBP. Only 12% (*n* = 17) of the invited employees were excluded or refused to participate in the study. Therefore, we consider that the external validity is good.

Although Roland-Morris Disability Questionnaire is rather insensitive to change when symptom level is low [56], active interventions were still effective in reducing long-term physical impairment. Respective effect sizes were between medium and large (Table 5).

Our main results emphasise the use of simple, low back specific questionnaire as an early screening instrument and the effectiveness of active, biopsychosocial exercise interventions (Rehab and Physio) for employees that are still working but have reported moderate level LBP symptoms. The main effects were consistent, long-lasting and were also supported by the results of the secondary outcomes. Together with our previous RCT [21], we suggest that early management of LBP is feasible for this employee group in the OH setting.

## Conclusions

Employees at risk of chronic and progressing LBP can be identified by using a simple health survey. The work ability of these individuals will be supported effectively in the occupational health service by offering them active, early phase LBP rehabilitation.

In this study, active interventions resulted in long-term reduction of several LBP related symptoms and improvement in quality of life over 2 years when compared to natural course. Yet, the cost-effectiveness of these interventions should be evaluated. Future research should also address the question of whether the same intervention approach is effective in different industries and health care settings.

## Abbreviations

15-D: 15-Dimensional health related quality of life questionnaire
Advice: Self-care advice by an OH physician intervention arm
ANOVA: Analysis of variance
CI95%: 95% confidence interval(s)
Control: Natural course of LBP, control group for the interventions
FABQ: Fear Avoidance Beliefs Questionnaire
FABQ_ph_: Fear Avoidance Beliefs Questionnaire, physical activity subscale
FABQ_w_: Fear Avoidance Beliefs Questionnaire, work subscale
ICD-10: International Statistical Classification of Diseases and Related Health Problems, 10th revision
LBP: Low back pain
OH: Occupational health
OSW: Oswestry Disability Questionnaire (sum value)
PHI: Physical impairment
Physio: Progressive back exercises intervention arm
QoL: Quality of life
RCT: Randomised controlled trial
Rehab: Multidisciplinary rehabilitation in the hospital outpatient clinic intervention arm
RM-18: Roland-Morris Disability Questionnaire 18 item scale (0–18)
VAS: Visual Analogue Scale (0–100 mm), pain intensity measurement
